# Dose to organs in the supraclavicular region when covering the Internal Mammary Nodes (IMNs) in breast cancer patients: A comparison of Volumetric Modulated Arc Therapy (VMAT) versus 3D and VMAT

**DOI:** 10.1371/journal.pone.0205770

**Published:** 2018-10-19

**Authors:** Vishruta A. Dumane, Richard Bakst, Sheryl Green

**Affiliations:** Department of Radiation Oncology, Icahn School of Medicine at Mount Sinai, New York, New York, United States of America; North Shore Long Island Jewish Health System, UNITED STATES

## Abstract

During breast/chest wall and regional nodal irradiation (RNI), standard 3D conformal techniques can fail to meet the dosimetric constraints for the heart and ipsilateral lung. VMAT can improve the dosimetric sparing of the heart and lungs. However the unnecessary increase in dose to the organs in the supraclavicular region as a result of using VMAT can be avoided. In this work we investigate potential dosimetric advantages of combining 3D with VMAT to improve sparing of these organs. Ten breast cancer patients requiring radiation therapy to the breast/chest wall and RNI including the IMNs, and who did not have a viable 3D conformal plan were chosen for the study. Each patient was planned with VMAT and with a combination of 3D for the supraclavicular region and VMAT for the breast/chest wall followed by a dosimetric comparison. Prescription dose was 50.4 Gy in 28 fractions. For similar coverage to the PTV and IMNs, doses to the esophagus and cord were reduced by 17.8 Gy and 15.5 Gy while mean dose to the thyroid and larynx were also reduced by 16.5 Gy and 11.7 Gy respectively. Maximum brachial plexus dose was the same in both techniques. The ipsilateral lung V_20Gy_ increased by 3.1% but was still < 30%. No significant differences were noted in doses to the heart, total lung and contralateral breast. However V_5Gy_ to the contralateral lung was reduced by 8.5% with the combined plan. Using 3D conformal planning for the supraclavicular region and VMAT over the breast/chest wall improves sparing of the esophagus, cord, thyroid and larynx while reducing low dose exposure to the contralateral lung and does not compromise doses to the heart, ipsilateral lung and total lung.

## Introduction

The role of regional nodal irradiation (RNI) during postmastectomy radiotherapy (PMRT) to the chest wall reduces locoregional recurrence distant metastases and improves overall survival [1, 2]. With the recent publication of two randomized trials that showed improvement in disease-free survival in 1–3 node-positive breast cancer patients receiving whole breast irradiation (WBI) after breast conserving surgery (BCS) along with RNI is going to increase the likelihood of inclusion of the internal mammary nodes (IMNs) in the treatment volume [3, 4]. Depending on the anatomy, standard 3D conformal planning techniques may not suffice to produce a treatment plan that is able to adequately spare the heart and the ipsilateral lung while irradiating the target volume that includes the IMNs. Partially wide tangents (PWT) and photon/electron (P/E) matching are commonly used 3D conformal techniques; with the former being the most appropriate balance between target coverage and normal tissue sparing [5,6] although the P/E technique can be useful in certain situations when depth of the lung included in the tangential field is > 3 cm [7].

Irrespective of the technique, the requirement to irradiate the IMNs increases exposure to the nearby critical organs especially the heart and the ipsilateral lung. For certain unfavorable anatomies, VMAT has been shown to be useful in reducing dose to the heart and lungs while generating more conformal isodose distributions [8]. However, there is also an increase in low dose exposure to non-target tissue. Specifically, organs in the supraclavicular region can be spared from this unnecessary exposure. At our institution, we have used VMAT to treat patients who are unable to have a viable 3D conformal plan. However, patients are seen to develop symptoms of grade 2–3 esophagitis within the first 2 weeks of treatment. 3D conformal planning over the supraclavicular region typically consists of an oblique field that does not enter/exit through structures medial to the supraclavicular node. Therefore a combination of 3D conformal planning over the supraclavicular region and VMAT over the breast/chest wall is likely to reduce unnecessary exposure to organs in this region without compromising the advantage of VMAT in improved sparing of the heart and lungs over standard 3D conformal planning. In this work, we report on a dosimetric comparison of VMAT versus a combination of 3D and VMAT for breast cancer cases requiring radiation to the breast/chest wall and RNI including the IMNs.

## Materials and methods

### Patient selection

The Program for the Protection of Human Subjects at the Icahn School of Medicine at Mount Sinai is the ethics and Institutional Review Board (IRB) committee that approved this study. 10 breast cancer patients (5 left sided and 5 right sided) were retrospectively chosen for the study. Since this was a retrospective study, the need for informed consent was waived. These patients required RNI including the IMNs, however due to unfavorable anatomy, standard 3D techniques such as PWT or P/E were found to irradiate too much heart and/or the ipsilateral lung. The first 6 out of these 10 patients were planned and treated with VMAT alone. However, 2 of these patients (~33%) were found to develop grade 2–3 esophagitis within completion of 30%-50% of their treatment. It was hence decided to plan and treat the next 4 patients using a combination of 3D conformal planning over the supraclavicular region and VMAT over the breast/chest wall to better spare the esophagus as well as other structures medial to the supraclavicular node. Patients in this study had stage II-IV breast cancer. Two patients had undergone a bilateral mastectomy with one patient receiving bilateral tissue expanders. Five patients had unilateral mastectomy and the remaining were intact breast. 6 patients who were treated using VMAT alone were retrospectively planned with a combination of VMAT and 3D conformal planning and 4 patients who were treated with the combination were retrospectively planned with VMAT alone. Patients were simulated in the supine position on an angle board with both arms raised and head turned away to the contralateral side. CT scans were obtained at 3 mm intervals extending from the chin to the upper abdomen during free breathing.

### Target delineation

The clinical target volume (CTV) consisted of the chest wall/breast tissue, axillary level I, II, III, supraclavicular and IMNs and were done as per published guidelines [9]. The planning target volume (PTV) was formed by adding a 5 mm margin to the CTV in all directions and included the skin in the chest wall region for mastectomy cases. This margin was provided to account for respiratory motion and setup errors. For intact breast cases, the first 5 mm of the skin was excluded from the PTV. Critical organs contoured were the heart, ipsilateral lung, contralateral lung, total lung and contralateral breast/implant where applicable. The energy used for all the plans was 6 MV. Dose prescribed was 50.4 Gy in 28 fractions. Dosimetric constraints are outlined in Table 1.

**Table 1:** Dose constraints.

Structure	Parameter	Constraint
PTV	D_95_ (%) V_95_ (%) D_05_ (%)	≥ 95 ≥ 95 ≤ 115
IMN	D_95_ (%)	≥ 90
Ipsilateral lung	V_20Gy_ (%)	≤ 30
Heart	Mean (Gy)	≤ 8

### VMAT planning

VMAT plans were generated using 2 coplanar arcs. To decide upon the geometry of these arcs, first, the gantry angle at which the projected separation of the PTV in the beam’s eye view (BEV) was found to be the largest was chosen. Since treatment volumes tend to be large and due to limitations of MLC leaf travel, on certain LINACS as being 15 cm, coverage of the PTV had to be split into two arcs. These arcs overlapped at the isocenter by 2 cm and were within a 200° range. The collimator angle was kept at 0°. Details regarding the field arrangement have been published elsewhere [8]. All treatment plans were performed with the Eclipse treatment planning system version 13.6 (Varian Medical Systems, Palo Alto, USA). The anisotropic analytical algorithm (AAA) was used for dose calculation. The optimization algorithm used was the Progressive Resolution Optimizer (PRO). During optimization, priority was given to cover 95% of the IMNs with at least 90% of the prescription dose (i.e 45 Gy) or more while achieving D_95_, V_95_ ≥ 95% and D_5_ ≤ 115% for the PTV, followed by mean heart dose (MHD), ipsilateral lung V20 Gy and dose to the contralateral lung and breast/implant.

### VMAT and 3D conformal planning

The target volume superior to the supraclavicular junction was planned using a single off-cord oblique supraclavicular field with appropriate blocking of the cord, esophagus and other structures medially while covering the supraclavicular node. The humeral head was blocked laterally. The energy used was 6 MV and the prescription depth was chosen accordingly such that nodal areas within the supraclavicular field were covered by at least 45 Gy. VMAT planning was used to cover the PTV inferior to the junction over the heart and the ipsilateral lung. Matching of the superior and inferior plans was accomplished using the single isocenter half beam block technique [10, 11] with asymmetric jaw. The collimator angle was kept at 0° for both the superior and inferior plans so that to prevent beam divergence into each other. The PTV for optimization purposes in this VMAT plan (PTV_OPT) was defined such that the superior-most slice was 6 mm inferior to the matchline/supraclavicular junction. This was done to avoid hotspots at the junction while planning with VMAT. On the other hand to avoid areas of underdosing at the junction, the slices of the PTV in between the level of the isocenter and the superior-most slices of the PTV_OPT were constrained in the optimizer to receive at least 45 Gy. This dose was determined iteratively such that the hotspot at the junction was limited to ≤ 105%. The VMAT plan was optimized such that priority was given to cover 95% of the IMNs with at least 90% of the prescription dose or more while achieving D_95_ and V_95_ of the combined plan to be ≥ 95% and the D_5_ at ≤ 115% for the PTV followed by mean heart dose (MHD), V_20Gy_ of the ipsilateral lung and dose to the contralateral lung and breast/implant.

### Dosimetric evaluation

Dose volume histograms (DVH) were generated and the dosimetric parameters collected for plans for each patient were compared. Statistical analysis was performed in MATLAB using the Wilcoxon signed-rank test at a significance level of ≤ 0.05. This test is a non-parametric hypothesis test used when comparing two related samples and does not assume the population to be normally distributed.

## Results

Dosimetric comparison of critical organs namely the spinal cord, brachial plexus, esophagus, thyroid and larynx between VMAT versus VMAT + 3D is shown in Table 2, while comparison of PTV and IMN coverage, doses to the heart, ipsilateral lung, total lung, contralateral lung and contralateral breast/implant are shown in Table 3.

**Table 2:** Comparison of critical organ dose namely the spinal cord, brachial plexus, esophagus, thyroid and larynx for VMAT versus VMAT + 3D.

Structure	Parameter	VMAT	VMAT +3D	*P* value
Cord	Maximum (Gy)	28.0±1.9	12.5±2.0	<0.01
Brachial Plexus	Maximum (Gy)	56.4±1.1	55.2±1.5	NS
Esophagus	Maximum (Gy)	45.0±2.2	27.2±4.5	0.03
	Mean (Gy)	9.0±0.6	6.3±0.5	<0.01
Thyroid	Mean (Gy)	30.1±2.9	13.6±2.9	<0.01

**Table 3:** Dosimetric comparison of PTV coverage, IMN coverage and critical organ doses namely the heart, ipsilateral lung, total lung, contralateral lung and contralateral breast for VMAT versus VMAT + 3D.

Structure	Parameter	VMAT	VMAT+3D	*P* value
PTV	D_95_ (%)	97.0±0.6	96.4±0.4	NS
	V_95_ (%)	96.3±0.6	96.1±0.3	NS
	D_5_ (%)	113.2±1.2	113.3±0.9	NS
IMN	D_95_ (%)	97.8±1.3	96.3±1.1	NS
Heart	Mean (Gy)	6.5±1.5	5.5±0.6	NS
	V_25Gy_ (%)	0.7±0.3	0.6±0.3	NS
	V_15Gy_ (%)	4.0±1.4	3.9±1.4	NS
	V_5Gy_ (%)	39.0±9	40.2±8.3	NS
Ipsilateral Lung	Mean (Gy) V_20Gy_ (%) V_10Gy_ (%) V_5Gy_ (%)	15.3±0.3 24.7±0.7 46.9±1.7 84.3±3.1	16.4±0.5 27.8±1.2 49.2±1.6 84.9±2.8	0.01 0.02 NS NS
Contralateral	Mean (Gy)	3.6±0.4	3.2±0.3	0.02
Lung	V_10Gy_ (%)	4.8±1.7	2.7±1.2	NS
	V_5Gy_ (%)	24.4±3.5	15.9±3.2	0.04
Contralateral Breast	Mean (Gy)	4.2±0.2	4.1±0.2	NS

**Fig 1:**
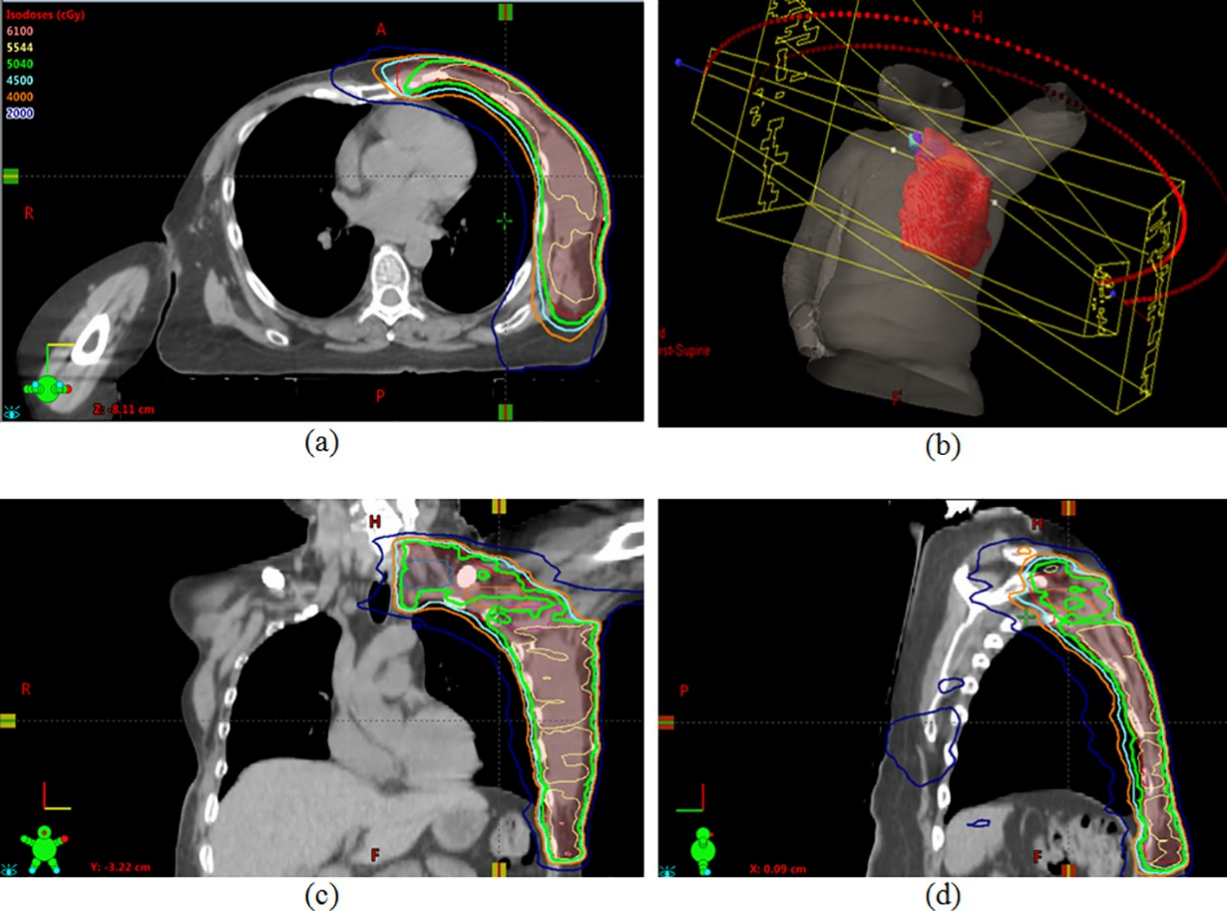
**Dose distribution in the axial (a), coronal (c) and sagittal (d) planes using only VMAT to cover the PTV, while (b) shows the field arrangement**.

**Fig 2:**
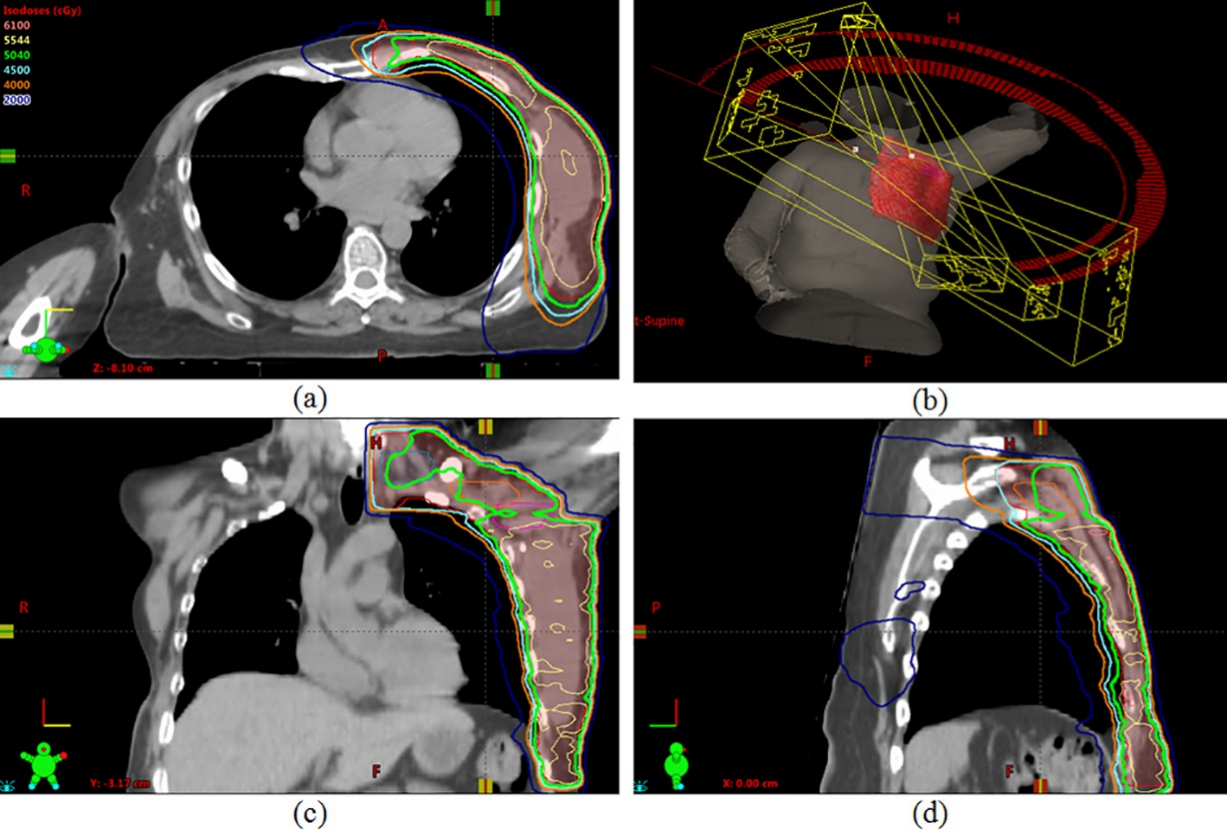
**Dose distribution in the axial (a), coronal (c) and sagittal (d) planes using a combination of VMAT over the breast/chest wall region and 3D conformal planning with an off-cord oblique to cover the supraclavicular area, while (b) shows the field arrangement**.

**Fig 3:**
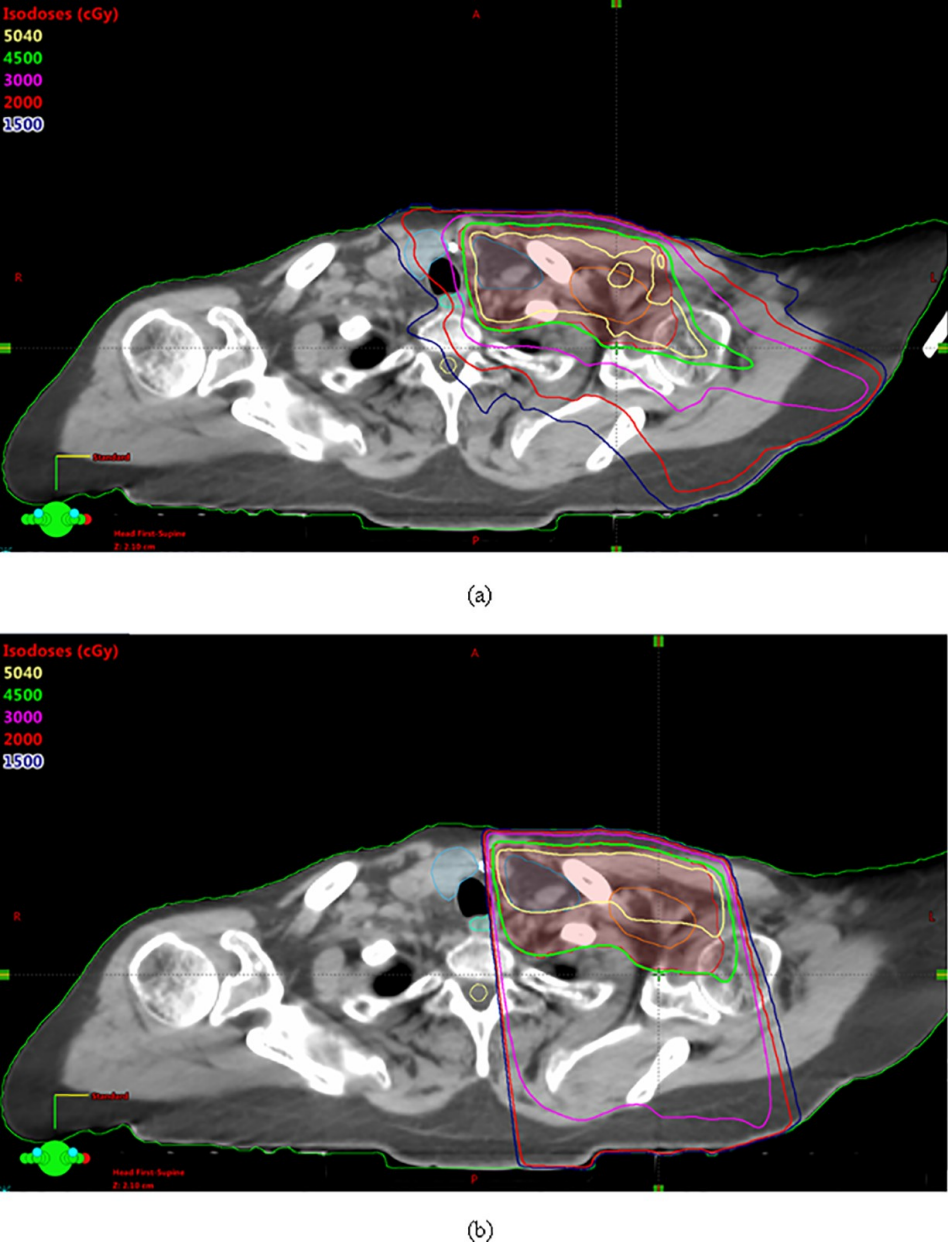
**Dose distribution in the axial plan for the VMAT only plan (a) and the combination of VMAT + 3D (b)**.

Dose distribution using only VMAT has been shown in Fig 1. Two partial coplanar arcs, one rotating in the clockwise and the other in the counterclockwise direction to cover the PTV are used for this plan. The arcs overlap at the isocenter by 2 cm and are 200° in range. The collimator angle used is 0°. Dose distribution using a combination of VMAT over the breast/chest wall region and 3D conformal planning with an off-cord oblique to cover the supraclavicular area is shown in Fig 2. Fig 3 shows a comparison of the dose distribution in the supraclavicular region in the axial plane between VMAT alone versus VMAT + 3D. The plan was done such that at least 45 Gy covered the supraclavicular PTV and nodes. Higher volumes of the esophagus, thyroid and the spinal cord are being covered by isodose lines lower than this prescription dose, namely 30 Gy, 20 Gy and 15 Gy in the VMAT plan. However in the combination plan, where a single off-cord oblique is used, lower volumes of these structures are exposed to these doses.

### PTV coverage

Differences in D_95_ and V_95_ for the PTV as well as D_95_ for the IMN were insignificant as were the differences in D_5_ for the PTV.

### Critical organs in the supraclavicular region

Using the combination of VMAT and 3D, the maximum dose to the esophagus was significantly reduced by 17.8 Gy and that to the cord by 15.5 Gy over using VMAT alone. Mean esophagus, thyroid and larynx doses were also reduced by 2.7 Gy, 16.5 Gy and 11.7 Gy respectively. No difference was noted in maximum dose to the brachial plexus.

### Heart

The mean heart dose (MHD) was lower with the combination of VMAT and 3D conformal planning by 1 Gy, however this was not statistically significant. Significant differences were also not noted in the V_25Gy_ or in the volumes of the heart covered by lower doses such as V_15Gy_ and V_5Gy_ between the two techniques.

### Ipsilateral lung

The ipsilateral lung V_20Gy_ was increased with the combination of VMAT and 3D by 3.1% and the mean ipsilateral lung dose was also slightly raised by almost 1 Gy compared to using VMAT alone. These differences were found to be statistically significant. Volumes of the ipsilateral lung covered by lower doses namely V_10Gy_ and V_5Gy_ showed no significant difference between the two planning techniques.

### Total lung

The difference in mean total lung dose for combination of VMAT and 3D versus VMAT alone was < 0.5 Gy and that in the V_20Gy_ was < 1.5%. However, no significant differences in volumes of the total lung covered by lower doses namely V_10Gy_ and V_5Gy_ were noted.

### Contralateral lung

The difference in mean contralateral lung dose was < 0.5 Gy between the two techniques. However, the V_5Gy_ to the contralateral lung was reduced by 8.5 Gy on using a combination of VMAT and 3D, a result found to be statistically significant.

### Contralateral breast

Mean contralateral breast dose was 4.2 Gy for VMAT and 4.1 Gy for combination of VMAT and 3D and was not significantly different between the two techniques.

## Discussion

The choice of the appropriate planning technique when treating the IMNs strongly depends on the patient’s anatomy [5–8]. The PWT and P/E techniques are commonly used standard 3D conformal planning techniques, with the former considered to be a good balance of target coverage and dose to the heart and ipsilateral lung [6]. When a 3D conformal planning technique is able to meet the desired dosimetric constraints, is a preferable treatment planning and delivery technique because it uses fewer gantry angles and avoids increase in low dose to the patient. Each of the cases in our study had either a mean heart dose (MHD) ≥ 10 Gy and/or the ipsilateral lung V20 Gy ≥ 35% when using 3D conformal planning. It is understood that radiation to the heart is unavoidable for left-sided cases and inclusion of the IMNs only further increases this exposure and likelihood of developing ischemic heart disease in long term. The rate of a major coronary event (MCE) increases linearly with MHD at the rate of 7.4% per Gy [12]. Similarly, exposure to the ipsilateral lung cannot be ignored.

Studies in patients who received RT to the IMNs while maintaining the ipsilateral lung V_20Gy_ < 30% showed ~6% grade 1 and 2 radiation pneumonitis (RP) and no incidence of grade 3 and 4 RP [13–15]. However, for patients in whom the ipsilateral lung V_20Gy_ was increased to around 35%, the grade 1 and 2 complications were higher at 23% and 11.5% respectively. With 3D conformal planning, the concern tends to be the rather larger volumes of heart and ipsilateral lung covered by higher doses. Due to the use of multiple arcs/projections with VMAT, doses 30 Gy and higher are better conformed to the target volume while being carved around the heart and the ipsilateral lung [8]. This reduces volume of the heart and ipsilateral lung receiving 30 Gy or more compared with standard techniques, thus improving their sparing and reducing the risk of MCE and RP after RT. Due to increased use in projection angles for beam delivery, there is increase in low dose exposure with VMAT, which is unavoidable over the heart and ipsilateral lung. However, increase in exposure to other organs by the supraclavicular region such as the esophagus, spinal cord, larynx and thyroid is unnecessary. At our institution we noted that some patients started developing symptomatic esophagitis within 2 weeks of treatment. One way to mitigate this was to keep the standard 3D conformal planning technique to treat the supraclavicular region using an off-cord oblique field while utilizing VMAT only over the heart and ipsilateral lung where it was required to improve sparing. This helped reduce the mean and/or maximum dose to the esophagus, spinal cord, larynx and thyroid significantly without compromising sparing of the heart and lung achieved with VMAT inferior to the supraclavicular junction. Although the ipsilateral lung V_20Gy_ was ≤ 30% in each case, on average it was slightly increased when 3D conformal planning was used to plan the supraclavicular region. This is because when planning using VMAT in this region, the isodose lines 20 Gy or higher are more conformal around the target volume, thereby sparing the lung as opposed to a single field which irradiates the entire lung in the field with at least 20 Gy.

The correlation between volume of heart covered by low dose such as 1 to 2 Gy from radiation therapy for breast cancer and the incidence of heart disease has been investigated in the literature [16]. Results indicate that there are no perfusion defects or cardiac function defects as a result of this low dose exposure. No worsening of these defects within a 1 year of exposure has been found either. In our study, with VMAT, we were able to reduce the MHD on average from 10 Gy with 3D conformal planning to ≤ 6.5 Gy using VMAT or a combination of VMAT for the breast/chest wall and 3D for the supraclavicular region, reducing the risk of MCE from radiotherapy in the long term for the patient. However, it is still desirable to keep the low dose exposure to the heart as low as possible while maintaining adequate coverage to the target because of the absence of long term follow-up data from treatments with VMAT for breast cancer. In addition to the heart, low dose to the lung also needs to be minimized. Studies indicate that total dose, dose per fraction and volume of irradiated lung influence the amount of lung morbidity that is caused from RT [17, 18]. In patients who received concurrent chemotherapy for esophageal cancer, the V_5Gy_ of the contralateral lung was noted to be an important predictor of radiation pneumonitis (RP) [19]. Contralateral lung V_5Gy_ ≥ 58% was associated with symptomatic RP ≥ grade 2. In our study, no significant differences were seen in low dose to the ipsilateral lung and total lung between VMAT versus combination of VMAT and 3D. Although the contralateral lung V_5Gy_ was much lower in our study with both the techniques, it was still lower with the combination plan by almost 9%. This improved sparing of the contralateral lung is expected because of the absence of entrance and exit dose from VMAT arcs into the contralateral side in the supraclavicular area that is covered with a single oblique field.

Maximum dose to the brachial plexus showed no difference between the two techniques. This was because part of the brachial plexus was within the PTV below the supraclavicular junction covered by VMAT. Dose to the contralateral breast was not compromised on using the combination plan because most of the contralateral breast tissue in the superior-inferior direction was located by the region covered by VMAT.

## Conclusions

In situations when 3D conformal planning is unable to meet dosimetric constraints for the heart and ipsilateral lung, VMAT can help meet the dose constraints for these structures. A combination of VMAT over the breast/chest wall and 3D conformal planning over the supraclavicular region can further help reduce unnecessary exposure to the esophagus, cord, thyroid and larynx without compromising on the dosimetric advantage already attained with the VMAT plan.

## Supporting information

S1 Data DATA PLOS ONE.xlsx. VMAT VERSUS VMAT+3D.
(XLSX)
